# Preparation and Characterization of Phenolic Foam Modified with Bio-Oil

**DOI:** 10.3390/ma11112228

**Published:** 2018-11-09

**Authors:** Yuxiang Yu, Yufei Wang, Pingping Xu, Jianmin Chang

**Affiliations:** College of Materials Science and Technology, Beijing Forestry University, 35 Qinghua East Road, Haidian District, Beijing 100083, China; yuyuxiang0612@163.com (Y.Y.); yufei_wang@126.com (Y.W.); m18813102213@163.com (P.X.)

**Keywords:** phenolic resin, bio-oil, phenolic foam, toughness

## Abstract

Bio-oil was added as a substitute for phenol for the preparation of a foaming phenolic resin (PR), which aimed to reduce the brittleness and pulverization of phenolic foam (PF). The components of bio-oil, the chemical structure of bio-oil phenolic resin (BPR), and the mechanical performances, and the morphological and thermal properties of bio-oil phenolic foam (BPF) were investigated. The bio-oil contained a number of phenols and abundant substances with long-chain alkanes. The peaks of OH groups, CH_2_ groups, C=O groups, and aromatic skeletal vibration on the Fourier transform infrared (FT-IR) spectrum became wider and sharper after adding bio-oil. These suggested that the bio-oil could partially replace phenol to prepare resin and had great potential for toughening resin. When the substitute rate of bio-oil to phenol (B/P substitute rate) was between 10% and 20%, the cell sizes of BPFs were smaller and more uniform than those of PF. The compressive strength and flexural strength of BPFs with a 10–20% B/P substitute rate increased by 10.5–47.4% and 25.0–50.5% respectively, and their pulverization ratios decreased by 14.5–38.6% in comparison to PF. All BPFs maintained good flame-retardant properties, thermal stability, and thermal isolation, although the limited oxygen index (LOI) and residual masses by thermogravimetric (TG) analysis of BPFs were lower and the thermal conducticity was slightly greater than those of PF. This indicated that the bio-oil could be used as a renewable toughening agent for PF.

## 1. Introduction

Phenolic foam (PF) is increasingly used in building structural materials due to its good thermal isolation, high dimensional stability, and particularly outstanding flame-retardant properties (no dripping combustion, low flammability, low smoke density and smoke toxicity) [1,2,3,4,5,6]. However, the application of PF is severely restricted by its high brittleness and pulverization, which is related to the lack of flexible functional groups in its chemical structures [2,4,5]. Thereby, a great number of research efforts have focused on increasing the toughness of PF to overcome its brittleness and pulverization.

Toughening of PF can be summarized into two methods: Physical modification and chemical modification. The physical modification focuses on introducing external toughening agents, such as inert fillers [7,8,9] and chopped fibers [3,10,11], into PF by physical blending. Chemical modification is a technique that concentrates on introducing flexible long chains into the molecular chain of PF by a chemical reaction, which has attracted extensive attention due to its notable toughening effect [2,12,13,14]. Chemical toughening agents, such as polyurethane [2,14], polyethylene glycol [4,12], and polyether [13], have been widely used to toughen PFs. However, considering the high price of the modifiers discussed above, modifiers from renewable natural compounds, such as lignin [6,15,16], tannin [17,18], and cardanol [1], have been a focus of research in recent years.

Bio-oil is a promising and inexpensive liquid product derived from the fast pyrolysis of renewable biomass [19,20,21]. Due to the abundant phenolic compounds of bio-oil, extensive inquiries have been conducted into using bio-oil as a fossil phenol substitute in the synthesis of bio-oil phenolic resin (BPR) [22,23,24]. There are two ways of substituting bio-oil for fossil phenol: (i) Using the pyrolytic lignin fractionated from bio-oil [22], and (ii) using the whole bio-oil [23,24]. The use of the whole bio-oil is attractive because it avoids the separation of pyrolytic lignin, and the aldehydes in bio-oil can also react with phenols to improve the degree of polymerization [23]. In addition, the compounds in the bio-oil with long flexible chains, such as guaiacol, can react with the reactive phenol hydroxyl or methylol groups of phenolic resin (PR) to introduce the long flexible chains into PR, which further improves the toughness of PF. Therefore, partial substitution of phenol with whole bio-oil to prepare PF can not only ease the dependence on petroleum and improve the price competitiveness, but also toughen PF. However, few examples in the literature have reported about the toughness modification of PF by whole bio-oil.

The objective of the study was to develop a foaming BPR by a reaction of bio-oil, phenol, and paraformaldehyde under alkaline conditions. The components of bio-oil were tested by gas chromatographic-mass spectrometric (GC-MS) analyses. The characterizations of BPRs were determined by Fourier transform infrared spectrometry (FT-IR). Bio-oil phenolic foams (BPFs) with different substitute rates of bio-oil to phenol (B/P substitute rate) were prepared. The basic characteristics, microstructure, flammability, thermal isolation, and thermal stability of BPFs were investigated by a universal test, polarizing microscope, limited oxygen index (LOI), and thermogravimetric (TG) analysis.

## 2. Materials and Methods

### 2.1. Materials

Bio-oil was obtained by fast pyrolysis of *Larix gmelinii* (Rupr.) Kuzen. in a fluidized bed at 550 °C for 2–3 s, which is the biomass residence time at the Lab of Fast Pyrolysis of Biomass and Productive Utilization (Beijing Forestry University, Beijing, China). Phenol, paraformaldehyde, and sodium hydroxide (NaOH) were purchased from Beijing Chemical Industries Reagent Co., Ltd., Beijing, China. Petroleum ether, tween-80, *p*-toluenesulfonic acid, and phosphoric acid were provided by Xilong Chemical Reagent Co., Ltd., Guangdong, China.

### 2.2. Preparation

#### 2.2.1. Synthesis and Characterization of BPRs

Foaming BPRs with different B/P substitute rates (0, 10, 20, and 30 wt.%) were synthesized at the molar ratio of phenol (include bio-oil)/paraformaldehyde/NaOH, which was 1:2:0.4. Firstly, phenol and 75 wt.% NaOH solution (40 wt.%) were added into a 250 mL three-necked flask. Afterwards, the 75 wt.% paraformaldehyde was slowly added to the flask and kept at 65–75 °C for 20 min. The mixture was heated to 90 °C and held for 30 min. Secondly, the residual 25 wt.% paraformaldehyde, 25 wt.% NaOH solution (40 wt.%), and bio-oil were added and remained at 80 °C for 60 min. Thirdly, the pH value of the mixture was adjusted to between 6.8 to 7.2 using hydrochloric acid (37 wt.%), and the mixture was rapidly cooled down to 40 °C in 20 min to yield resin. The resultant resins were denoted as PR, 10%BPR, 20%BPR, and 30%BPR.

The viscosity of the resin was tested by an NDJ-5S rotating viscometer (CANY, Shanghai, China) at 25 °C. The solid content and curing time of resin were determined according to China National Standards (GB/T 14074-2013). In order to accurately determine the curing time of BPR in the foaming process, composite curing agents (*p*-toluenesulfonic acid/phosphoric acid) and the test temperature (75 °C) of the foaming process were used. In this test, 50 ± 0.1 g of resin and 6 ± 0.01 g of curing agent were stirred well at room temperature. The mixture (10 ± 0.1 g) was then placed in a test tube at 75 °C and the time until the stirring bar could not move was recorded. Each test above was repeated at least three times. The characterization of BFRs are summarized in Table 1.

#### 2.2.2. Preparation and Characterization of BPFs

Based on the weight of resin, 5 wt.% surfactants (Tween-80), 8 wt.% blowing agents (petroleum ether), and 12 wt.% composite curing agents (*p*-toluenesulfonic acid/phosphoric acid was 2:1) were added into the BPR and rapidly mixed well at room temperature. Then the mixture was poured into a mold and bubbled at 75 °C for 40 min. These prepared foams were denoted as PF, 10%BPF, 20%BPF, and 30%BPF.

The apparent density of foam was tested based on the China National Standard (GB/T 6343-2009). The compressive strength and flexural strength of the foam were measured with a universal testing machine (Insrton, Havisham, England) according to the China National Standard (GB/T 8813-2008 and GB/T 8812-2007). A LOI test of the foam was carried out using a JF-3 oxygen index meter (Jiangning Analysis Instrument Co., Jiangsu, China) according to the China National Standard (GB/T 2406-2008). The thermal conductivity of foam was investigated by the LFA467 thermal conductivity testing instrument (Netzsch, Selb, German). The pulverization ratio was obtained based on China National Standard (GB/T 12812-2006). In this test, a weight of 200 g was put on the sample (30 × 30 × 30 mm^3^). Then, the sample was pushed back and forth on a 300 mesh abrasive paper 30 times at a constant force and the distance of each single-pass friction was 250 mm. The pulverization ratio was measured by the weight loss of a sample after friction [7]. At least five replicates were used for these tests.

### 2.3. Analysis

A gas chromatographic-mass spectrometric (GC-MS) analysis of bio-oil was recorded on a Shimadzu GC/MS-QP system (Kyoto, Japan). FT-IR analysis of the bio-oil and cured resin were obtained by a Nicolet iS5 FT-IR (Nicolet, Wisconsin, USA). The microstructure of the foam was observed using a WV-CP230/G polarizing microscope (Panasonic, Suzhou, China). The cell size distributions were calculated on ImageJ 1.47. Thermogravimetric (TG) analysis of the foam was examined at a heating rate of 10 °C/min using a Q5000IR analyzer (TA Instruments, the USA) under a nitrogen atmosphere.

## 3. Results and Discussion

### 3.1. Components of the Whole Bio-Oil

The moisture of bio-oil is 29.82%, and the main organic compounds of bio-oil characterized by GC-MS are displayed in Table 2. As seen, the phenols represent the major peak area (33.08%) and numerous substances in bio-oil had good reactivity with formaldehyde, such as phenol, cresols, guaiacol, and resorcinol. Besides, many phenols with long unsaturated alkane chains, such as guaiacol, could bring toughening groups into the molecular structure of resin when reacting with formaldehyde or reactive phenol hydroxyl and methylol groups of PR. Moreover, some ketones, aldehydes, esters, alcohols, and acids with long-chain alkanes in bio-oil also had toughening effects [25]. Furthermore, the aldehydes, like formaldehyde, acetaldehyde, and furaldehyde of bio-oil, could react with the unreacted phenol to reduce the free phenol and improve the polymerization of resin. Nevertheless, the low boiling point substance and esters of bio-oil could be used as blowing agent and surfactants respectively, which further reduced the cost of PR. These demonstrated that the bio-oil had great potential for partially replacing phenol to prepare resin while toughening resin. It is also important to note that the bio-oil contains a large number of acids (9.24%), resulting in a low pH value. Therefore, bio-oil should be added at the later stage of the synthesis process of PR to reduce the influence on the addition reaction [22].

### 3.2. FT-IR Analysis of BPRs

**Figure 1:**
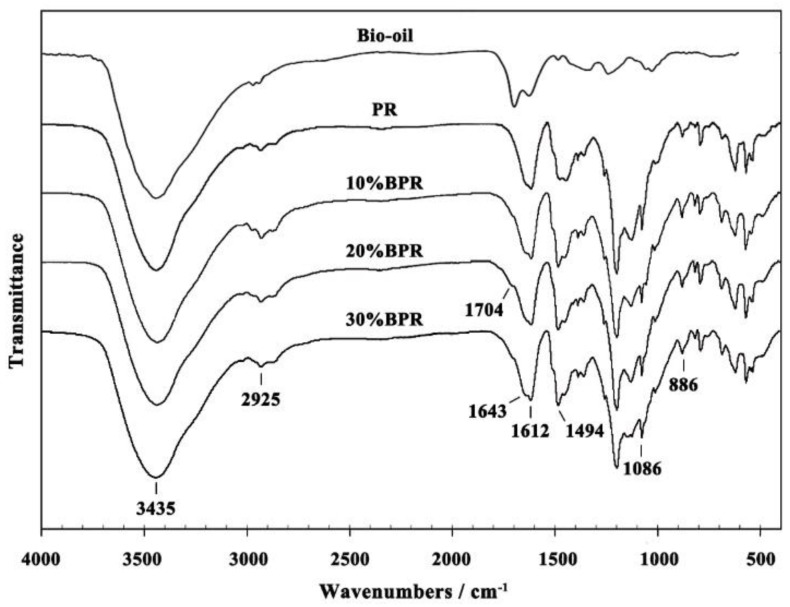
Fourier transform infrared (FT-IR) spectrum of cured PR and BPRs.

The FT-IR spectra of bio-oil and cured PR and BPRs are depicted in Figure 1, and the functional groups that correspond to the major peaks have been identified and listed in Table 3 [26,27]. As seen in Figure 1, the three large peaks of bio-oil at 3435 cm^−1^, 1704 cm^−1^, and 1612 cm^−1^ were assigned to the vibration of OH groups, C=O groups, and aromatic skeleton respectively. These peaks were ascribed to the phenol, ketone, aldehyde, and ester components in bio-oil, which was consistent with the GC-MS result of bio-oil in Part 3.1. Additionally, the peak of CH_2_ at 2925 cm^−1^ could prove the existence of long-chain alkanes in bio-oil.

When using the whole bio-oil to partly replace phenol, all of the prepared BPRs presented similar curves to that of the PR, indicating that the BPRs and PR had similar chemical structures. However, some differences between BPR and PR are also found in Figure 1, e.g., the new peak of C=O stretching vibration at the region of 1704 cm^−1^ appeared and became wider with the increase of the B/P substitute rate. Moreover, the peak at 1643 cm^−1^ assigned to C=O also became wider. These indicated more different compounds with C=O groups in resins after adding bio-oil. In other words, numbers of C=O groups were introduced into the chemical structure or formed during the sysnthesis of PR owing to the bio-oil. In addition, the stonger peaks of BPRs than PR at the peaks at 1612 cm^−1^ and 1494 cm^−1^ were assigned to the aromatic skeletal vibration, which indicated that the phenolic compounds in bio-oil were involved in the synthesis of resin. These meant that abundant flexible functional groups could be introduced into the chemical structure of PR with the reactions between bio-oil and polyformaldehyde or resin intermediate, such as the dimethylphenol and trimethylphenol. The introduction of flexible long chains could also be demonstrated by the increase of the CH_2_ peak after adding bio-oil. The peak of CH_2_ at 2925 cm^−1^ of BPRs, especially 10%BPR, was stronger than that of PR. Another possible reason for the larger CH_2_ peaks of BPRs was the facilitate impact of bio-oil on the balance of synthetic reaction. For example, the long-chain alkanes in the opposite site of the phenol hydroxyl in guaiacol made the electron cloud density on the benzene ring migrate to the side chain, thus improving the activation of C–O–C bonds to turn into the more stable CH_2_ bond [26]. These results indicated that adding bio-oil could not only toughen resin, but also improve the polymerization of resin. However, compared to 10%BPR and 20%BPR, the CH_2_ peak at 2925 cm^−1^ of 30%BPR decreased, suggesting that there was an optimum B/P substitute rate that best improved the polymerization of PRs.

### 3.3. Characteristics of BPFs

#### 3.3.1. Microstructure of BPFs

**Figure 2:**
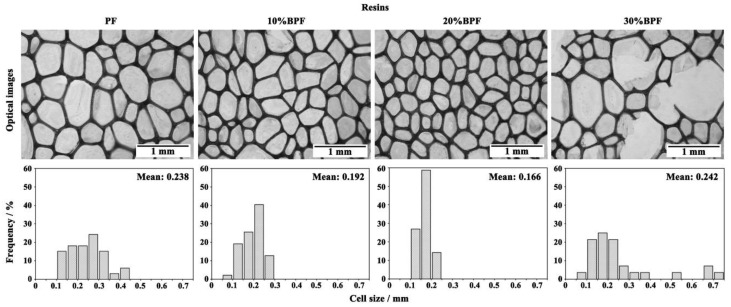
Optical images and cell size distribution of PF and bio-oil phenolic foams (BPFs).

The PF and BPFs were examined by an optical microscope and their cell size distributions were calculated in order to study the effect of the B/P substitute rate on the microstructure. As shown in Figure 2, the BPFs and PF were made up of a great number of closed cells. The mean cell sizes of 10%BPF and 20%BPF were 0.192 mm and 0.166 mm, respectively, which are 19–30% smaller than that of PF (0.238 mm). The smaller cell sizes might be due to the much larger molecular compounds in bio-oil and their dragging effect on long side chains [28,29], increasing the viscosity of resin (Table 1) and limiting the growing and merging of cells. Meanwhile, the cell sizes of 10%BPF and 20%BPF, particularly 20%BPF (0.10–0.25 mm), are more uniform than that of PF (0.10–0.45 mm). This was caused by the abundant low volatile compounds in bio-oil, which widened the boiling point range of the foaming agent in the forming progress. However, in the case of 30%BPF, the cell sizes were larger and less uniform. Furthermore, some fragments from the bubble collapses appear. These might be due to: (i) The longer curing time of 30%BPR (Table 1) leading to the failure of achieving the appropriate viscosity of resin in time to stop cells growing and merging [9], and (ii) the lower solids content (Table 1) and the decreased polymerization of 30%BPR (Figure 1).

#### 3.3.2. Basic Characteristics of BPFs

The apparent density, pulverization ratio, compressive strength, and flexural strength of PF and BPFs are summarized in Table 4. The apparent densities of 10%BPF and 20%BPF were higher than that of PF, which was due to the smaller and more uniform cell sizes. However, further increasing the B/P substitute rate decreased the apparent density. Compared with PF, the pulverization ratio of BPFs decreased first and increased with the increase of the B/P substitute rate. The pulverization ratio of 20%BPF dropped to its lowest point (8.9%) and decreased by 38.6% in comparison with PF. This was due to the improved toughness of BPFs because of the long side chains in bio-oil. However, with the B/P substitute rate increasing to 30 wt.%, the fragments from the foam collapses led to the higher pulverization ratio. With the increase of the B/P substitute rate, the compressive strength and flexural strength of BPFs first increased, maximizing at 20% B/P substitute rate, and then decreased. Compared with PF, the compressive strength and flexural strength of 20%BPF increased by 47.4% and 50.0%, respectively. These were mainly related to the more uniform cell sizes of 20%BPF than other foams. In addition, the improvement of toughness of 20%BPF by bio-oil also led to the higher compressive strength and flexural strength. However, further increasing the B/P substitute rate weakens the compressive strength and flexural strength owing to the cell collapses.

#### 3.3.3. Thermal Analysis

**Figure 3:**
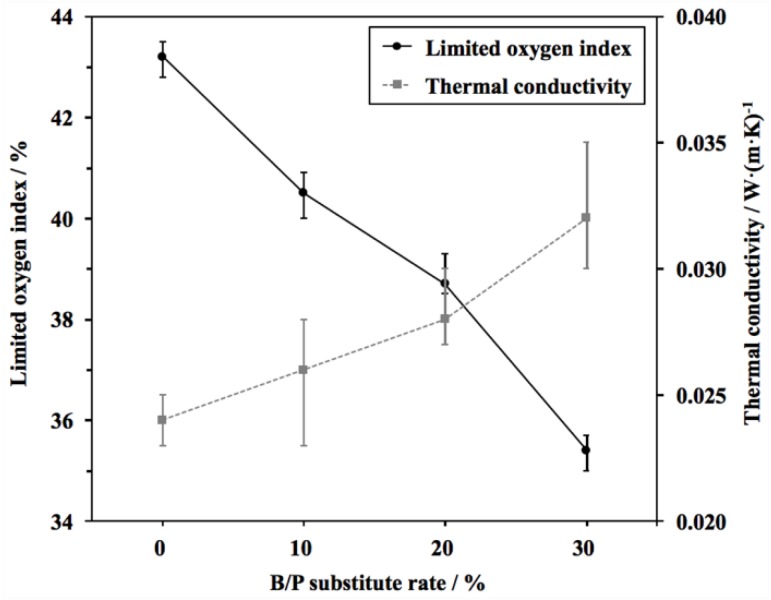
Limited oxygen index and thermal conducticity of PF and BPFs.

The PF is widely used because of its outstanding flame-retardant properties. However, a considerable amount of research reported that most of the toughing agents would deteriorate the flame resistance of PF [2,5]. Therefore, the LOI, thermal conductivity, and thermal stability of BPFs were investigated. As shown in Figure 3, the LOI value of PF foam is 43.2%, whereas the values of BPFs decrease to 40.5%, 38.7%, and 35.4%, respectively. It is well known that the benzene rings are difficult to burn owing to the easy charring when exposed to the flame [13]. Therefore, the weakened flame resistance of BPFs was due to the decrease of the benzene rings on the backbone chains by using bio-oil as a substitute for phenol. Fortunately, all the LOI values of BPFs were larger than the B1 standard value (≥30%) according to the China National Standards (GB 8624-2012), which meant that the BPFs still maintained good flame-retardant property. The thermal conductivity of foams was little affected by the B/P substitute rate and the difference in value between BPFs and PF only ranges from 0.002 W/(m·K) to 0.008 W/(m·K). The increased thermal conductivity of BPFs was due to the substances with low thermal conductivity in bio-oil. Additionally, the cell collapses could be another reason for the increased thermal conductivity of 30%BPF.

**Figure 4:**
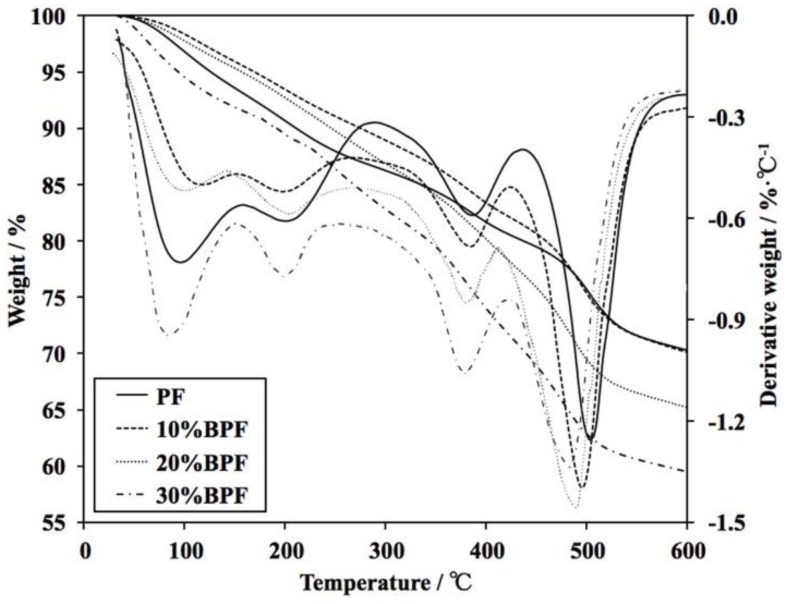
Thermogravimetric (TG) and derivative thermogravimetric (DTG) curves of PF and BPFs.

The TG and derivative thermogravimetric (DTG) curves of PF and BPFs under a nitrogen atmosphere are illustrated in Figure 4, and the relevant degradation data of PF and BPFs are addressed in Table 5. Compared with PF, the initial degradation temperatures (*T*_−5%_, the temperatures at 5% weight loss) [2,4] of 10%BPF and 20%BPF were slightly higher, which indicated that the incorporation of 10–20% bio-oil improved the thermal stability of foams at lower temperatures. The reason was related to the thick cell walls of 10%BPF and 20%BPF (Figure 3), which made it more difficult for the water and volatiles to evaporate. However, the *T*_−5%_ of 30%BPF decreased. The maximum weight loss temperature (*T*_max_) of the BPFs slightly shifted to a lower temperature with an increasing B/P substitute rate, and the residual masses at 600 °C of BPFs were lower than that of PF. These were due to the decrease of the benzene rings in modified foams because of the replacement of bio-oil to phenol. However, all the residual masses at 600 °C of BPFs were nearly above 60%. The decline proportion of BPFs was only 0.2–14.9% in comparison with PF, which meant that the BPFs still maintained good thermal stability.

## 4. Conclusions

Whole bio-oil had great potential for replacing phenol and toughening resin because of its numbers of phenols and abundant substances with long-chain alkanes. Using bio-oil to partly replace phenol introduced abundant flexible functional groups into the chemical structure of PR. This was proved by the smaller pulverization ratio, larger compressive strength, and flexural strength of 10%BPF and 20%BPF in comparison with PF. Adding bio-oil also made the cell sizes of foams smaller and more uniform. These indicated that bio-oil had a positive impact on the toughness of foams. However, the decrease of LOI and residual masses at 600 °C, as well as the light increase of thermal conducticity of BPFs, suggesting that the bio-oil, like most toughing agents, deteriorated the flame resistance of PF. Fortunately, the negative effect of bio-oil was slight and the BPFs still maintained good flame-retardant property, thermal isolation, and thermal stability. Therefore, the bio-oil could be used as a renewable toughening agent for PF.

## Figures and Tables

**Table 1:** Basic characteristics of phenolic resin (PR) and bio-oil phenolic resin (BPR).

Resins	Viscosity (25 °C, mPa·s)	Solids Content (%)	Curing Time (75 °C, s)
PR	1743 ± 67	79.8 ± 0.4	698 ± 29
10%BPR	2852 ± 35	80.3 ± 0.3	823 ± 32
20%BPR	3889 ± 48	78.6 ± 0.3	1062 ± 43
30%BPR	3205 ± 81	74.7 ± 0.5	1605 ± 35

**Table 2:** Identification and quantification of the main organic components in bio-oil by gas chromatographic-mass spectrometry (GC-MS).

Compounds	Molecular Formula	Peak Area (%)
**Phenols**		33.08
Phenol	C_6_H_6_O	4.23
Cresols	C_7_H_8_O	3.59
Catechol	C_6_H_6_O_2_	1.11
Guaiacol	C_7_H_8_O_2_	2.67
4-methylcatechol	C_7_H_8_O_2_	2.50
2-methoxy-4-methylphenol	C_8_H_10_O_2_	3.14
4-ethylresorcinol	C_8_H_10_O_2_	1.84
4-ethylguaiacol	C_9_H_12_O_2_	1.14
3,4-dimethoxyphenol	C_8_H_10_O_3_	2.10
Eugenol	C_10_H_12_O_2_	1.27
4-allyl-2,6-dimethoxyphenol	C_11_H_14_O_3_	1.80
Other phenols		7.68
**Ketones**		17.68
Hydroxyacetone	C_3_H_6_O_2_	4.08
2-butanone	C_4_H_8_O	1.94
4-hydroxyacetophenone	C_8_H_8_O_2_	1.46
Acetovanillone	C_9_H_10_O_3_	1.22
2,4-dimethoxyacetophenone	C_10_H_12_O_3_	1.62
Other ketones		7.36
**Aldehydes**		11.18
Acetaldehyde	C_2_H_4_O_2_	4.91
2-Furaldehyde	C_5_H_4_O_2_	2.08
Vanillin	C_8_H_8_O_3_	1.27
Syringaldehyde	C_9_H_10_O_4_	0.86
Other aldehydes		2.07
**Sugars**		10.35
D-Mannose	C_6_H_12_O_6_	6.53
*β-D-*lactose	C_6_H_12_O_6_	1.22
Other sugers		2.60
**Acids**		9.24
Acetic acid	C_2_H_4_O_2_	2.97
4-hydroxybenzoic acid	C_7_H_6_O_3_	1.84
Homovanillic acid	C_9_H_10_O_4_	1.22
4-methylnonanoic acid	C_10_H_20_O_2_	1.17
Nonadecanoic acid	C_19_H_38_O_2_	0.94
Other acids		1.10
**Esters**		6.75
Methyl acetate	C_3_H_6_O_2_	1.94
Ethyl methacrylate	C_6_H_10_O_2_	1.03
Ethylene glycol diacetate	C_6_H_10_O_4_	1.59
Octyl acetate	C_10_H_20_O_2_	1.20
Other esters		1.00
**Alcohols**		5.89
Ethylene glycol	C_2_H_6_O_2_	1.03
Furfuryl alcohol	C_5_H_6_O_2_	1.60
4-hydroxychroman	C_9_H_10_O_2_	1.17
Heneicosyl alcohol	C_21_H_44_O	0.94
Other alcohols		1.15
Others		5.81
**Total**		100.00

**Table 3:** Peaks and assignment of FT-IR spectra for cured PR and BPRs.

Wave Number (cm^−1^)	Vibration	Assignment
3435	ν (OH)	Phenolic OH and aliphatic OH stretching vibration
2925	ν (CH_2_)	Aliphatic CH_2_ asymmetric stretching vibration
1704, 1643	ν (C=O)	(Phenolic) C=O stretching vibration
1612, 1494	ν (C=C)	C=C aromatic ring stretching vibration
1086	ν (C–O–C)	Phenolic C–O–C stretching vibration

ν: Stretching vibration.

**Table 4:** Basic characteristics of PF and BPFs.

Foams	Apparent Density (kg·m^−3^)	Pulverization Ratio (%)	Compressive Strength (MPa)	Flexural Strength (MPa)
PF	49.2 ± 1.9	14.5 ± 0.4	0.19 ± 0.03	0.24 ± 0.03
10%BPF	53.2 ± 1.3	12.4 ± 0.2	0.21 ± 0.04	0.30 ± 0.04
20%BPF	58.1 ± 1.6	8.9 ± 0.2	0.28 ± 0.05	0.36 ± 0.02
30%BPF	45.6 ± 0.9	13.6 ± 0.3	0.16 ± 0.02	0.19 ± 0.02

**Table 5:** Degradation data of PF and BPFs by TG analysis.

Foams	*T*_−5%_ (°C)	*T*_mas_ (°C)	Residue at 600 °C (%)
PF	126	505	70.27
10%BPF	169	495	70.10
20%BPF	156	489	65.22
30%BPF	103	482	59.79
